# Eight-step method to build the clinical content of an evidence-based care pathway: the case for COPD exacerbation

**DOI:** 10.1186/1745-6215-13-229

**Published:** 2012-11-29

**Authors:** Cathy Lodewijckx, Marc Decramer, Walter Sermeus, Massimiliano Panella, Svin Deneckere, Kris Vanhaecht

**Affiliations:** 1 Respiratory Department, University Hospitals Leuven, Leuven, Belgium; 2 Department of Public Health, KU Leuven University of Leuven, Leuven, Belgium; 3 European Pathway Association, Kapucijnenvoer, Leuven, Belgium; 4 Faculty of Medicine, KU Leuven University of Leuven, Leuven, Belgium; 5 Department of Public Health, Department of Clinical and Experimental Medicine, Faculty of Medicine, Amedeo Avogadro University of Eastern Piedmont, Novara, Italy; 6 Western Norway Research Network on Integrated Care, Helse Fonna, Haugesund, Norway

**Keywords:** Critical pathway, Evidence based medicine, Standardization, Cluster randomized trial, Chronic obstructive pulmonary disease

## Abstract

**Background:**

Optimization of the clinical care process by integration of evidence-based knowledge is one of the active components in care pathways. When studying the impact of a care pathway by using a cluster-randomized design, standardization of the care pathway intervention is crucial. This methodology paper describes the development of the clinical content of an evidence-based care pathway for in-hospital management of chronic obstructive pulmonary disease (COPD) exacerbation in the context of a cluster-randomized controlled trial (cRCT) on care pathway effectiveness.

**Methods:**

The clinical content of a care pathway for COPD exacerbation was developed based on recognized process design and guideline development methods. Subsequently, based on the COPD case study, a generalized eight-step method was designed to support the development of the clinical content of an evidence-based care pathway.

**Results:**

A set of 38 evidence-based key interventions and a set of 24 process and 15 outcome indicators were developed in eight different steps. Nine Belgian multidisciplinary teams piloted both the set of key interventions and indicators. The key intervention set was judged by the teams as being valid and clinically applicable. In addition, the pilot study showed that the indicators were feasible for the involved clinicians and patients.

**Conclusions:**

The set of 38 key interventions and the set of process and outcome indicators were found to be appropriate for the development and standardization of the clinical content of the COPD care pathway in the context of a cRCT on pathway effectiveness. The developed eight-step method may facilitate multidisciplinary teams caring for other patient populations in designing the clinical content of their future care pathways.

## Background

Standardization of the clinical care process through integration of evidence-based knowledge has proven to be an effective strategy for reducing unwanted variations in treatment and for minimizing the probability of medical errors [1]. However, major difficulties arise when introducing evidence and clinical guidelines into routine daily practice, and many patients, as a result, do not receive appropriate care, or receive unnecessary or harmful care [2-5].

A possible tool to facilitate implementation of evidence into practice is a care pathway. Care pathways are complex interventions for mutual decision making, organization, and standardization of predicTable care for a well-defined group of patients during a well-defined period [6-8]. One of the active ingredients in care pathways is the integration of a set of evidence-based key interventions [8,9].

Care pathways induce change at different levels of the organization (that is, patient, team, hospital); consequently, variability at individual level outcomes may reflect the impact of higher-level complexity processes. To deal with these multilevel effects, cluster randomized designs are strongly recommended when studying the impact of care pathways [10,11]. Importantly, in cluster randomized controlled trials (cRCTs) the care pathway under evaluation is implemented at different sites. Consequently, a challenge within cRCT designs is to standardize the intervention in order to deliver the ‘same’ intervention at the different sites under study [10,12-14]. Standardization in complex interventions refers to adaptation of the care pathway components to the context level, without compromising the integrity of the intervention being evaluated across multiple sites [10,14,15].

In 2009, the European Pathway Association (E-P-A) launched the European Quality of Care Pathways (EQCP) study, an international cRCT addressing the impact of a care pathway for chronic obstructive pulmonary disease (COPD) exacerbations [9]. In the context of the EQCP study, the clinical content of a model COPD care pathway - implementable at the different experimental sites - needed to be developed, including a set of clinically applicable evidence-based key interventions and a set of reliable process and outcome indicators. This paper describes the development of the clinical content of a care pathway for in-hospital management of COPD exacerbation.

## Methods

**Figure 1:**
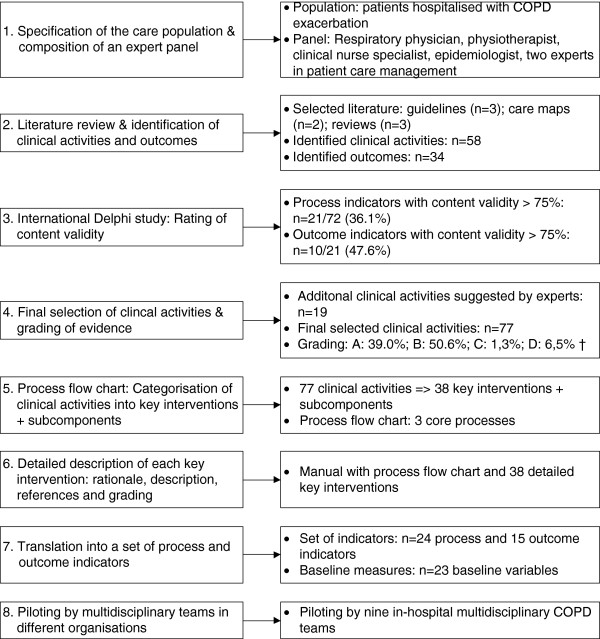
Eight-step method for development of the clinical content of an evidence based care pathway: the case for COPD exacerbation.

The clinical content of an evidence-based care pathway for COPD exacerbation was developed based on the process design methodology developed by Berry *et al.*[16], and the guideline development methods of the American College of Chest Physicians (ACCP) [17], the World Health Organization (WHO) [18] and the Healthcare Infection Control Practices Advisory Committee (HICPAC) [19]. Subsequently, based on the experiences of the COPD case, a generalized eight-step method for development of the clinical content of an evidence based care pathway was designed (Figure 1). This study was approved by the ethical committee of the University Hospitals Leuven as previously published in this journal [9].

## Results

A set of 38 evidence-based key interventions and a set of 24 process and 15 outcome indicators were developed in eight different steps. Both sets are displayed in Additional file 1 and Additional file 2, respectively. In the following section, description and rationale for each development step is presented.

### Step 1: Selection of the care population and selection of an expert panel

The patient population under study was specified as ‘Patients hospitalized with COPD exacerbation’. To ensure clinical validity and feasibility of the end product, an expert panel was involved in each step of the development method. This panel was composed of the following: (i) three clinicians with internationally recognized clinical and scientific expertise in COPD exacerbations: a respiratory physician (MD) who is also president of the European Respiratory Society, a physiotherapist (TT) who specializes in pulmonary rehabilitation, and a clinical nurse specialist in COPD (CL); (ii) an epidemiologist (MP) who specializes in organization of primary and secondary chronic care; and (iii) two professors (WS, KV) in patient care management who have extensive clinical and scientific expertise in development and implementation of care pathways [8,18-21]. All six experts had extensive research experience.

### Step 2: Literature review and extraction of clinical activities

**Figure 2:**
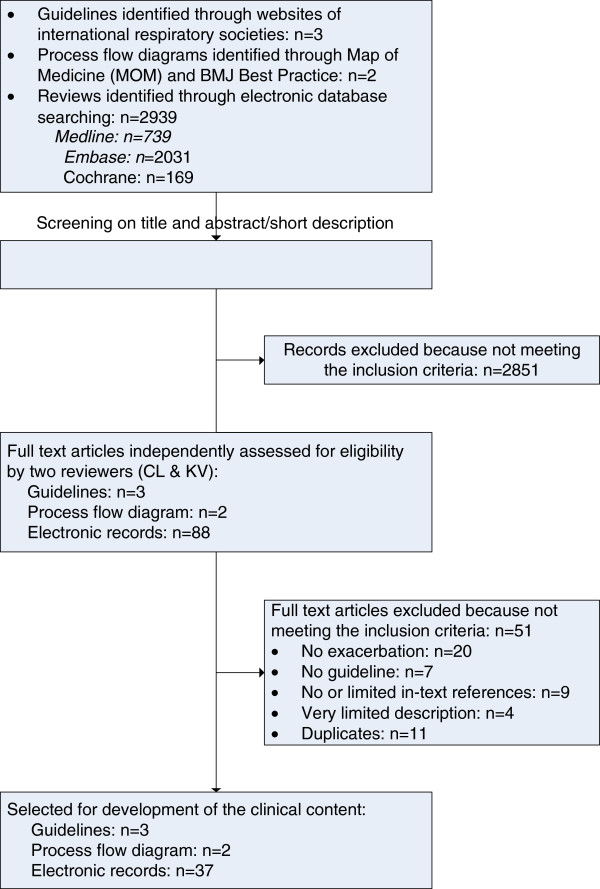
Literature search strategy.

To identify all available evidence for integration in the evidence-based COPD care pathway, an extensive literature review was conducted by the main researchers, CL and KV (Figure 2). First, an initial literature search was carried out in April 2008 in the context of the Delphi study, and an updated search was performed in June 2011. In the following section, the updated search is described [22].

The following resources were explored: (I) websites of international respiratory societies: American Thoracic Society (ATS) (http://www.thoracic.org); British Thoracic Society (BTS) (http://www.brit-thoracic.org.uk); European Respiratory Society (ERS) (http://www.ersnet.org); Global Strategy for Diagnosis, Management, and Prevention of COPD (GOLD) (http://www.goldcopd.org); National Institute for Health and Clinical Excellence (NICE) (http://www.nice.org.uk); Scottish Intercollegiate Guidelines Network (SIGN) (http://www.sign.ac.uk); (II) Public resources for evidence-based clinical practice guidelines (http://www. guideline.gov, http://www.g-i-n.net); (III) electronic databases including Medline and Embase and Cochrane; (IV) available process flow diagrams founded on evidence-based medicine (http://www.mapofmedicine.com, http://group.bmj.com/products/evidence-centre.com).

For guidelines developed by international societies, only those guidelines were considered that were updated within the last five years. For PubMed and Cochrane, we used the MeSH terms ‘COPD’ combined with (i) ‘practice guideline’, (ii) ‘disease exacerbation and patient care management’, and (iii) ‘outcomes’. For Embase, we used the MeSH terms ‘chronic obstructive lung disease’ combined with (i) ‘practice guideline’ and (ii) ‘disease exacerbation and patient care’, and (iii) ‘outcomes’. Non-MeSH terms used in Embase were ‘COPD’ in combination with ‘exacerbation and management’. Search limit parameters included: (i) published between 2005 and 2011, and (ii) written in English, French, German, Italian or Dutch.

Second, we performed a two-phase screening evaluation of publications selected from websites of the respiratory societies, Map of Medicine and the electronic databases. In the first phase, publications were appraised for relevance based on appropriateness of the title and abstract. If relevance was unclear, or if the abstract was unavailable, the publication was included for further appraisal of the full text. In the second phase, two independent researchers (CL and KV) reviewed the full text of the selected guidelines, reviews or process flow diagrams. The following inclusion criteria were used: (i) reportage of clinical processes and outcomes regarding in-hospital management of COPD exacerbation; (ii) evidence was reported in terms of guidelines, process flow diagrams, reviews or overview papers; (iv) published between 2005 and 2011; (v) published in English, French, German, Italian or Dutch; and (vi) quality of underlying evidence can be appraised by in-text references. The literature research revealed initially three guidelines, one process flow diagram, and 2,939 digital records from the electronic medical databases (Figure 2). After exclusion of irrelevant publications (n = 2,851), and after appraisal of full text, three guidelines, two process flow diagrams, and 37 reviews were included for development of the evidence-based clinical content of the COPD care pathway [5,23-56].

Finally, the selected literature was thoroughly screened for identification of all possible clinical activities and outcomes related to in-hospital management of COPD exacerbation. The detected clinical activities were extracted and listed, and the corresponding literature sources were recorded. In total, 58 different clinical activities were extracted from the selected literature (Table 1, no. 1–58). Besides these, 34 outcome categories were identified (Table 2).

**Table 1:** Clinical activities for management of patients hospitalized with COPD exacerbation

1. Medical history before exacerbation: prior measures of lung function (B)*	37. Smoking cessation advice when active smoker (A)
2. Medical history before exacerbation: spirometric classification of severity (B)	38. Appropriate prescription of short-acting bronchodilatators (A)
3. Medical history before exacerbation: documenting frequency and severity of attacks of breathlessness (B)	39. Appropriate prescription of long-acting bronchodilatators (β-agonists and/or anticholinergics) (A)
4. Medical history before exacerbation: documenting frequency and severity of chronic cough (B)	40. Appropriate prescription of inhaled corticosteroids (A)
5. Medical history before exacerbation: history of chronic sputum production (B)	41. Appropriate prescription of glucocorticosteroids: oral or intravenous (A)
6. Medical history before exacerbation: documenting possible limitation of daily activities (B)	42. Appropriate prescription of methylxanthines (theophylline or aminophylline) (A)
7. Medical history before exacerbation: prior arterial blood gas measurements in sTable condition (B)	43. Antibiotics in patients if indicated (A)
	44. Patient education information about recognition and treatment of exacerbation (A)
8. Medical history before exacerbation: number of previous exacerbations in the previous year (B)	45. Patient education: instruction on how to use inhalers (A)
9. Medical history before exacerbation: number of previous hospitalizations (B)	46. Chest physiotherapy: sputum clearance (A)
	47. Referral to pulmonary rehabilitation (A)
10. Medical history before exacerbation: pre-existing co-morbidities (A)	48. Monitoring of fluid balance (A)
	49. Fluid administration in dehydrated patients (A)
11. Medical history before exacerbation: present treatment regimen (A)	50. Supplementary nutrition in patients with BMI <20 (B)
	51. Screening and update of vaccination status (B)
12. Medical history before exacerbation: smoking status (B)	52. Deep venous thrombosis prophylaxis (A)
13. Medical history before exacerbation: sleeping and eating difficulties (B)	53. Treatment of co-morbid conditions (A)
14. Assessment of symptoms: physical examination (B)	54. Initiation of long-term oxygen therapy (LTOT) if the patient remains hypoxemic (A)
15. Assessment of differential diagnosis (B)	55. Assessment of medical discharge criteria (D)
16. Assessment of co-morbidities (B)	56. Assessment and management of home situation (A)
17. Temperature (B)	57. Oral information and discharge letter regarding prescribed home therapy and follow-up appointment (B)
18. Pulse rate (B)	58. Arrangement of follow-up appointment four to six weeks after discharge (D)
19. Blood pressure (B)	59. Medical history before exacerbation: number of previous admissions to ICU (D)
20. Alertness (B)	60. Medical history before exacerbation: cardiovascular status (B)
21. Skin color (B)	61. Glucose monitoring (B)
22. Pulse oximetry (D)	62. CT THORAX: 1 X year (B)
23. Arterial blood gas measurement: At admission (B)	63. ECHO CARDIO: 1 X year (B)
24. Arterial blood gas measurement: prior to discharge in patients hypoxemic during a COPD exacerbation (B)	64. Patient education: information about the nature of COPD (A)
25. Arterial blood gas measurement: in the following three months in patients hypoxemic during a COPD exacerbation (D)	65. Patient education: self-management plan (A)
26. Arterial blood gas measurement: after discharge in patients with long term oxygen therapy (LTOT) (B)	66. Patient education strategies for minimizing dyspnoea (A)
27. Chest X-ray (B)	67. Patient education information about oxygen treatment (A)
28. ECG (B)	68. Physiotherapy: breathing techniques (A)
29. Blood examination: hematology (B)	69. Physiotherapy: Activities of Daily Life (A)
30. Blood examination: biochemical tests (B)	70. Physiotherapy: positioning (A)
31. Blood examination: theophylline level in patients on theophylline therapy at admission (B)	71. Identification for pulmonary rehabilitation determinant (B)
32. Sputum culture and anti-biogram (B)	72. Body mass index (BMI) determinant (A)
33. Spirometry during hospitalization (not earlier than Day 3 because of acute condition) (C)	73. Screening for weight loss (A)
34. Admission to ICU if exacerbation is life threatening (B)	74. Referral to dietician in patient with obesity or cachexie (B)
	75. Assessment and management of anxiety and depression (B)
35. Controlled oxygen therapy in hypoxemic patients (A)	76. Information letter for general practitioner (B)
36. Assisted ventilation if necessary (A)	77. Discharge checklist (B)

**Table 2:** Identified outcomes for in-hospital management of COPD exacerbation

· Readmission: 30-day, 3-month, 6-month, 1-year	· Inhaled β-agonist therapy is required no more frequently than every four hours
· Number of hospital admissions	
· Interval before next admission	· Patient, if previously ambulatory, is able to cope with basic needs in his/her situation, in usual environment
· Frequency and severity of exacerbation	
· Mortality: in-hospital, 30-day, 3-month, 6-month, 1-year	· Patient is able to eat and sleep without frequent awakening by dyspnoea
· Survival: 1-year	
· Length of stay (LOS)	· Patient has been clinically sTable for 12 to 24 hours
· Level of understanding of inhaler therapy	· Last measure of arterial blood gases (ABGs) were accepTable according to condition of the patient
· Compliance with home oxygen therapy	
· Performance of physical exercise	· Patient and/or home caregiver fully understands correct use of therapy: oral medication therapy, inhaler therapy, oxygen therapy if home oxygen therapy
· Smoking status: 30-day, 3-month, 6-month, 1-year	
· Symptoms of anxiety and depression	
	Patient, family, and physician are confident that the patient can manage successfully
· Health-related quality of life (HRQL): symptoms, disability, morbidity and quality of life; psychological well-being)	· Lung function parameters: forced expiratory volume in one second (FEV_1_), forced vital capacity (FVC), inspiratory capacity
· Health status	
· Quality-adjusted life expectancy measure (QALY) and disability adjusted life years (DALY)	
	· Quality of sleep
· Functional capacity	· Nutritional status
· Exercise capacity	· Patients’ perception of coordination between hospital and home healthcare
· Physical performance: 6-minute walking distance (6-MWD), 20-MWD, shuttle walk test, maximum workload, treadmill time, maximum oxygen uptake, quadriceps strength, hand grip force, maximal inspiratory mouth pressure	
	· Patient satisfaction with therapy and care
	· Adverse event related to regular clinical examination by an investigator
· Severity of breathlessness: dyspnea, symptoms at rest and during exercise	· Cost of illness (COI) analysis
	· Absenteeism

### Step 3: International Delphi study for rating of content validity

Content validity was rated for 72 process and 21 outcome indicators by conducting an international Delphi study with a panel composed of 35 medical professionals from 15 countries. This panel consisted of 19 medical doctors, 8 nurses and 8 physiotherapists. The detailed methodology and the results of this study were published elsewhere [57-60].

In summary, panelists were asked to rate the relevance for follow-up of the process and outcome indicators in care pathways for COPD exacerbations. Consensus was defined as agreement by at least 75% of the panel members that an indicator is relevant for follow-up. Consensus was reached for 26 of 72 process indicators (36.1%) and 10 of 21 outcome indicators (47.6%). Highest consensus was reached for the process indicators for oxygen therapy (100%), pulmonary rehabilitation (100%), and patient education (94.5 to 88.6%), and for the outcome indicators for understanding of therapy (91.4 to 85.7%) and self-management (88.6 to 88.2%) [60].

### Step 4: Final selection of the clinical activities and grading of evidence

First, the list of 58 extracted clinical activities (step 2), together with the Delphi results (step 3), were sent to the clinical experts of the panel (MD, TT and CL) with a request to complete two tasks: (i) to review the 58 identified activities for validity and feasibility; and (ii) if indicated, to propose any additional clinical activity they believe is essential for in-hospital management of COPD exacerbations and which is lacking in the current activity list of clinical activities. Second, a consensus meeting was held with the entire expert panel in order to make a final selection of the clinical activities. As a result, all 58 clinical activities were appraised to be valid and feasible. In addition, 19 clinical activities beyond the 58 original ones were included (Table 1, nos. 59–77). Interestingly, for almost all these additional clinical activities, a more or less comprehensive description was available in the guidelines for management of stable COPD [27,32,61].

Finally, the strength of the evidence for the final 77 clinical activities was graded, so that clinicians know how much confidence they can place on the clinical recommendations included in the clinical care pathway [62]. The grading was performed by the clinical nurse specialist (CL) using the SIGN approach [63]. The grading approach of SIGN was chosen because this grading system is very transparent and provides a simplistic grading of evidence [62-64]. Importantly, if the level of evidence could not be derived based on the literature selected in step 2, an additional literature search was performed in Medline, Embase and Cochrane. Search terms included ‘COPD’ and key words related to the particular key intervention. Primarily, the search for additional evidence was focused on reviews performed according to standard criteria for reviews [22]. If not available, an additional search for clinical trials was conducted. Subsequently, two other clinical experts of the panel (MD and TT) checked the final grading. As a result, 30 activities were graded as evidence for level A (39.0%), 41 activities as level B (53.2%), 1 activity as level C (1.3%), and 5 as level D (6.5%) (Table 1).

An extensive list of care activities was generated by following the above-mentioned steps. However, providing such an exhaustive list of 77 care activities to the multidisciplinary teams would likely not encourage them to use this evidence in practice. Therefore, the next two steps were specifically undertaken to distil the list of care activities to a set of key interventions that would be useable and manageable in clinical practice.

### Step 5: Clustering of clinical activities into key interventions and categorization into process flow diagram

First, the 77 clinical activities were clustered into key interventions with subcomponents, based on the following criteria: (i) clinical activities are inextricably linked to each other (that is, measurement of basal metabolic index, advice on malnutrition, supplementary nutrition and so on were clustered into ‘nutrition’); (ii) clinical activities need to be performed by a specific team member (that is, breathing exercises, positioning and so on were categorized under physiotherapy); (iii) clinical activities need to be performed at a specific time point or within a specific time span of the care process (that is, activities regarding discharge management). As a result, the 77 clinical activities were clustered into 38 key interventions, with 9 of them comprising 2 to 15 subcomponents.

Second, the key interventions were categorized into three core processes (diagnostic, pharmacological and non-pharmacological management), and subsequently presented by means of a process flow diagram. In addition, within each of three core processes, key interventions were grouped into care blocks based on the overall content of these key interventions (for example, education, ventilation). The process flow diagram with the 38 key interventions is displayed in Additional file 1.

### Step 6: Detailed description of the key interventions

**Figure 3:**
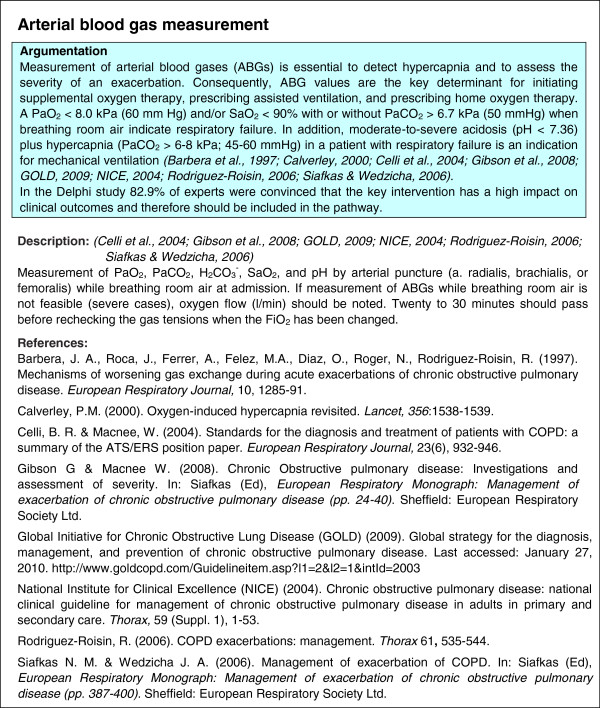
Example of detailed description of a key intervention: arterial blood gas measurement.

For each key intervention, the following components were included in the detailed description: (i) rationale, which addresses why it is of crucial importance that the key intervention is performed, and which describes expected impact on patient outcomes; (ii) description, which defines the exact content of the key intervention; (iii) in-text references and reference list; and (iv) grading of evidence. An example of a detailed description of a key intervention on arterial blood gas measurements is provided in Figure 3. In order to search for detailed information on the description and the rationale, selected publications and their reference list were explored. Second, information from the additional literature search, performed to establish level of evidence (step 4), was included.

### Step 7: Translation into a set of process and outcome indicators

Besides the set of key interventions, a set of process and outcome indicators needed to be developed to verify compliance to key interventions and to follow up the impact on outcomes. First, to select the final set of indicators, the expert panel convened for a consensus meeting. The selection process was based on the (updated) literature search (step 2), the Delphi survey (step 3), and the developed set of 38 evidence-based key interventions (step 5) [60]. As a result, a set of 24 process and 15 outcome indicators was developed, which are displayed in Additional file 2. The 24 process indicators include measurements on performance of diagnostic, pharmacological and non-pharmacological interventions. The 15 outcome indicators include measurements on readmission, mortality, length of stay (LOS), understanding of inhaler therapy, compliance with home oxygen therapy, performance of physical exercise, smoking status, anxiety and depression, health-related quality of life, management at home, functional status, self-reported health condition, medical consumption and an economic evaluation. On the basis of their expertise, the panel also selected a set of 15 baseline variables, including medical, socioeconomic, demographic and COPD-specific data.

Subsequently, the selected indicators and baseline variables were operationalized into objective measurements [65]. Based on the guidance of the Agency for Health Care Research and Quality (http://www.qualitymeasures.ahrq.gov) and the Joint Commission (http://www.jointcommission.org), each indicator and baseline variable was defined in an indicator protocol by the main researcher (CL). This process included defining of description, rationale or relation to quality, type of indicator (process, outcome, baseline), nominator and denominator, data collection method, data elements, data reporting (that is, proportion, relative proportion), criteria to meet expected outcomes, and references. An example of an indicator description is detailed in Additional file 3. Subsequently, the indicator protocol was mailed to the entire expert panel with a request to appraise each indicator description thoroughly for accuracy and feasibility. A meeting with the entire expert panel was convened to discuss the feedback and finalize the indicator protocol.

### Step 8: Piloting by multidisciplinary teams

The set of 38 key interventions, and the set of 24 process and 15 outcome indicators, were piloted by nine Belgian experimental COPD teams in the context of the EQCP study [9]. The multidisciplinary teams included pulmonologists, nurses, physiotherapists, dieticians, social workers and occupational therapists. The piloting occurred in four phases. First, feasibility of data collection was evaluated during a clinical audit before pathway implementation. For this phase, 105 patients from 9 Belgium hospitals were included [9]. Mean age was 67 years (SD: 10.0), and 68.6% of the patients were male. Approximately half of the patients had severe COPD, 21% had moderate COPD and another 21.9% had very severe COPD. Overall, after data analyses we determined that data collection is feasible, and only minor adaptations with regard to the patient record analysis were included.

Second, during a workshop in which team members of all nine multidisciplinary COPD teams attended, the process flow diagram, including the 38 key interventions, was presented. Subsequently, all key interventions were extensively discussed. Third, the detailed set of key interventions was provided to the study coordinator of each hospital. We requested all members of the multidisciplinary COPD team to extensively review the key intervention set and subsequently to provide feedback within two weeks. As a result, the feedback given during the workshop and provided after extensive appraisal by all teams showed that teams were very enthusiastic about the process flow diagram and underlying key interventions. Moreover, they agreed by consensus that the set of key interventions was valid and applicable for use in their practice.

However, the teams provided four main remarks regarding: (i) usefulness of spirometry during exacerbation because results may be inaccurate due to the compromised condition; (ii) feasibility of referral to pulmonary rehabilitation with regard to condition of the patient and availability of a rehabilitation center; (iii) type of inhaler medications and device (nebulizer vs inhaler); and (iv) finally content, workload and feasibility of patient education. First, with regard to spirometry, no hard evidence about accuracy and, thus, usefulness of spirometric tests during exacerbation is available and, thus, no specific guidance on whether or not to perform spirometric tests could be provided to the teams. This issue was specifically emphasized in the detailed set of key interventions. Concerning pulmonary rehabilitation, all teams were convinced about the importance of referring patients to rehabilitation, and consequently, during the workshop some alternatives with regard to availability of a rehabilitation center were discussed. Finally, with regard to inhaler therapy and patient education, a teaching workshop was organized and education tools for COPD teams and ready-to-use patient leaflets were provided.

Finally, the nine multidisciplinary COPD teams implemented the set of key interventions as an active component of their care pathway for in-hospital management of COPD exacerbation in the context of the EQCP study [9]. Six months after the start of development and implementation of the care pathway, the nine teams had the opportunity to report experiences, barriers and successful actions during a workshop. One major difficulty in implementing the educational package into the daily work routine was reported. Overall, the teams confirmed validity and clinical applicability of the set of 38 key interventions.

## Discussion

A set of 38 evidence-based key interventions for in-hospital management of COPD exacerbation was developed (see Additional file 1) and, subsequently, piloted and validated by multidisciplinary COPD teams from nine different hospitals. This overall approval indicates that the applied strategy is appropriate for the development and standardization of the clinical content of an evidence-based care pathway. Second, a set of 24 process and 15 outcome indicators was also developed (see Additional file 2). The pilot study showed that the measurements on the indicators were feasible for the multidisciplinary teams and the patients; only some minor adaptations were required. Subsequently, based on our experience and what we have learned from the COPD case, we designed a generalized eight-step method (Figure 1), with the aim to guide and inspire teams caring for other patient groups in designing the clinical content of their future evidence-based care pathways.

It is important to note that designing the care pathway content according to the eight-step strategy is a time-consuming process, especially with regard to the Delphi survey (step 3) and pilot testing (step 8). However, results of the Delphi survey and piloting are essential to ensure that the key intervention set is widely, clinically applicable. This is especially important when conducting a cRCT, in which the ‘same’ care pathway intervention needs to be implemented by different teams at different sites and possibly in different countries [9]. Teams developing care pathways should carefully plan an implementation strategy and budget enough time in their project plan for proper development of the clinical content of their care pathway.

A surprising finding is that, based on review of the literature (Step 2), the Delphi study, and face-to-face expert opinion, advanced care planning was not included in the set of 38 key interventions. On one hand, this can be explained due to the focus on management of acute COPD exacerbation. On the other hand, it is essential that advanced care planning and end of life discussions are initiated in advance of a life threatening situation, which can arise after COPD exacerbation [66]. Therefore, we acknowledge that an additional key-intervention with detailed reference to and description of advance life care planning should be included in this key intervention set.

An important limitation in the current strategy is the lack of patient involvement [67]. Patients can bring a different perspective to the quality improvement process, as they are likely to prioritize different aspects of care compared to clinicians, including interpersonal and amenity aspects; for example, communication with healthcare staff and quality of the food, rather than the technical and clinical aspects [68]. We believe that patients, for instance, by contacting patient societies, should have been involved in three phases of the eight-step method: (i) step 4: Final selection of the clinical activities; (ii) step 7: Translation into a set of indicators; and (ii) step 8: Piloting of the final set of key interventions. Including patients in these phases could have provided extra activities and outcomes, important from the patient perspective. After implementation of the key interventions, it will be interesting to gather information on patient preferences and opinions by performing open interviews with the patient and relatives, or by performing walk-throughs together with the patient [69]. Also, when applying the evidence-based care intervention in daily practice, clinicians should ensure that each of their individual patients is involved in decision making [67]. In this context, it is also recommended to develop a patient version that includes a brief and understandable summary of the set of key interventions.

We believe that developing the clinical care pathway content by using this newly developed and validated eight-step method will facilitate adequate integration of evidence-based knowledge into daily practice. Since the beginning of the 1990s, evidence-based clinical practice guidelines for almost all domains of medicine have been available worldwide, accessible more recently via the Internet [4,70,71]. However, we see high variability in the integration of knowledge from evidence-based guidelines into daily practice [4,72]. Common barriers for integration of evidence-based knowledge are disagreement with the evidence; lack of outcome expectancy; lack of time; and available evidence, such as guidelines being unnecessarily complex, and thus not so directly applicable for clinical practice [4,72,73]. This eight-step methodology can facilitate translation of evidence-based knowledge into clinically applicable key interventions, which can overcome barriers and assist clinicians both in selecting the best treatment options and in delivering safe and effective care [4]. However, besides providing a set of detailed evidence-based key interventions, consideration of factors like culture (safety, commitment to do better in practice, peer norms); teamwork; skills management; communication; leadership alignment; and support will be critical to successfully integrate evidence into practice and improve the care process [74]. In this context, care pathways can be very effective tools, as they bring all these pieces together [8,25,75].

Finally, we want to emphasize the potential role of professional medical associations in clinical content development for evidence-based care pathways. Many national and international societies have extensive clinical and research experience in the patient population of their clinical field, comprise a global network of experts in the field, have funding available and, last but not least, have comprehensive understanding and experience in synthesizing evidence-based knowledge and making this knowledge usable for daily clinical practice. Therefore, we believe that professional societies could play a major role in developing the clinical content of future evidence-based care pathways, especially in terms of clinical support, expert networking and input of resources.

## Conclusion

The set of 38 key interventions and the set of process and outcome indicators were found to be appropriate for the development and standardization of the clinical content of the COPD care pathway in the context of a cRCT on pathway effectiveness. The developed eight-step method may facilitate multidisciplinary teams caring for other patient populations in designing the clinical content of their future care pathways.

## Abbreviations

ABGs: Arterial blood gases; ACCP: American College of Chest Physicians; ATS: American Thoracic Society; BMI: Body mass index; BTS: British Thoracic Society; COI: Cost of illness; COPD: Chronic obstructive pulmonary disease; cRCT: Cluster randomized controlled trial; DALY: Disability adjusted life years; E-P-A: European Pathway Association; EQCP: European Quality of Care Pathways; ERS: European Respiratory Society; FEV_1_: Forced expiratory volume in one second; FVC: Forced vital capacity; GOLD: Global Strategy for Diagnosis Management, and Prevention of COPD; HICPAC: Healthcare Infection Control Practices Advisory Committee; HRQL: Health-related quality of life; LOS: Length of stay; LTOT: Long-term oxygen therapy; NICE: National Institute for Health and Clinical Excellence; QALY: Quality-adjusted life expectancy measure; SIGN: Scottish Intercollegiate Guidelines Network; WHO: World Health Organization.

## Competing interests

The authors declare that they have no competing interests.

## Authors’ contributions

CL KV, WS, SD and MP contributed to the draft and the final version of the paper. MD supervised and was closely involved in the development of the clinical content of the care pathway intervention. MP, KV and WS have the scientific lead of the EQCP study. KV is international coordinator of the EQCP study. All authors have read and approved the final manuscript.

## Supplementary Material

Additional file 1 **Process flow diagram for in-hospital management of COPD exacerbation.** This Additional file displays a process flow chart including 38 key interventions that should be performed for every patient entering the hospital with COPD exacerbation. The key interventions are classified under three core processes: Diagnostic, Pharmacological and Non-pharmacological management. Click here for file

Additional file 2 **Set of Process and outcome indicators for in-hospital management of COPD exacerbation.** This Additional file displays a set of validated process and outcome indicators for audit of care for in-hospital management of COPD exacerbation. Click here for file

Additional file 3 **Example of description of an indicator.** This Additional file displays the detailed description of an indicator according to the guidance of the Agency for Health Care Research and Quality (http://www.qualitymeasures.ahrq.gov) and the Joint Commission (http://www.jointcommission.org). Click here for file
