# Supplementary material for: Eight-step method to build the clinical content of an evidence-based care pathway: the case for COPD exacerbation
Source: Trials. 2012 Nov 29;13:229. doi: 10.1186/1745-6215-13-229 (PMC3543249; doi:10.1186/1745-6215-13-229)
Supplement: Additional file 1 — Process flow diagram for in-hospital management of COPD exacerbation. This Additional file displays a process flow chart including 38 key interventions that should be performed for every patient entering the hospital with COPD exacerbation. The key interventions are classified under three core processes: Diagnostic, Pharmacological and Non-pharmacological management. [file 1745-6215-13-229-S1.pdf]

Additional file 1: Process flow diagram for in-hospital management of COPD exacerbation

DIAGNOSTIC ASSESSMENT

History and examination

1. History before exacerbation →
   - Prior measures of lung function
   - Spirometric classification of severity
   - Documenting frequency and severity of attacks of breathlessness
   - Documenting frequency and severity of attacks of cough
   - History of chronic sputum production
   - Documenting possible limitatoins of daily activities
   - Prior arterial blood gas measurements in stable condition
   - Number of previous exacerbations in the previous year
   - Number of previous hospitalisations because of COPD in the previous year
   - Number of admissions to ICU in the previous year
   - Cardiovascular status
   - Pre-existing comorbidities
   - Current treatment regimen
   - Smoking status at admission
   - Sleeping and eating difficulties
2. Assessment of symptoms: Physical examination
3. Assessment of differenial diagnosis
4. Assessment of comorbidities

5. Daily assessment of vital parameters →
   - Temperature
   - Pulse rate
   - Blood pressure
   - Pulse oxymetry
   - Alertness
   - Skin color

Diagnostic tests

7. Arterial blood gas measurement
8. Chest X-ray
9. Electrocardiogram

10a. Blood examination →
   - Hemaetology
   - Biochemical tests (i.e. blood glucose monitoring)
   - Theophylline level in patients on theophylline therapy at admission

10b. Glucose monitoring

11. Sputum culture and antibiogram
12. Spirometry
13. 1-yearly CT Thorax
14. 1-yearly Echo cardio

15. Admission to ICU if exacerbation is life treathening

PHARMACOLOGICAL MANAGEMENT

Inhaler therapy

- 16. Short-acting bronchodilators → Initiation, continuation or increase of doses and frequency
- 17. Methylxanthines → Consider if inadequate or insufficient response to short-acting bronchodilators
- 18. Long-acting bronchodilators → Initiation, continuation or increase of doses and frequency
- 19. Inhaled glucocorticoids → Initiation, continuation or increase of doses and frequency

Or combination therapy

Corticoids & Antibiotics

- 20. Oral prednisolone 30-40 mg during 7-14 days
- 21. Antibiotics <u>if indicated</u> →

<u>Indication:</u>

- Patients with increased sputum purulence, and also show increased dyspnoea and or increased sputum volume
- Patients who are critically ill and who need mechanical support
- Consolidation on a chest radiograph
- Clinical signs of pneumonia

NON-PHARMACOLOGICAL MANAGEMENT

Ventilation

22. Controlled oxygen therapy in patients hypoxemic during admission
23. Assisted ventilation if indicated

Education

24. Smoking cessation advice in active smokers
25. Patient education: COPD & self management
    - Nature of COPD
    - Recognition and treatment of exacerbations
    - Self management strategies
    - Strategies for minimizing dyspnoea
26. Patient education: Inhaler therapy
27. Patient education: Home oxygen therapy

Physiotherapy

28. Physiotherapy
    - Training / support in activities of daily life
    - Positioning
    - Chest Physiotherapy: airway clearance
    - Breathing techniques
29. Pulmonary rehabilitation
    - Identification for pulmonary rehabilitation
    - Referral to pulmonary rehabilitatoin

Other measures

30. Screening and updating of vaccinational status: influenza; pneumococcus
31. Nutritional status
    - Assessment:
      - □ BMI
      - □ Weight loss
    - Dietician:
      - □ Maltnutrition: nutritional supplement; advice
      - □ Overweight: advice
32. Deep venous thrombosis prophylaxis
33. Fluid balance
    - Monitoring of fluid balance
    - Fluid administration in dehydrated patients
34. Treatment of co-morbid conditions
35. Assessment and management of anxiety and depression

Discharge

36. Arterial blood gas measurement prior to discharge in patients hypoxemic during exacerbation
37. Prescription of home oxygen therapy in patients who remain hypoxemic at discharge from the ward
38. Discharge management
    - Assessment of medical discharge criteria
    - Assessment and management of home situation
    - Oral information and discharge letter regarding prescribed home therapy and follow-up appointment
    - Arrangement of follow-up appointment 4-6 weeks after discharge
      - □ The patient's ability to cope at home
      - □ Measurement of FEV1
      - □ Re-assessment of inhaler technique and understanding treatment regime
      - □ In severe COPD: need for LTOT* and/or home nebuliser usage
      - □ Advice on smoking cessation as necessary
      - □ Arterial blood gas measurement:
        - ○ In the following three months in patients hypoxemic during a COPD exacerbation
        - ○ After discharge with LTOT
    - Information letter for general practitioner
    - Discharge checklist

* LTOT= Long Term Oxygen Therapy
