# Supplementary material for: Eight-step method to build the clinical content of an evidence-based care pathway: the case for COPD exacerbation
Source: Trials. 2012 Nov 29;13:229. doi: 10.1186/1745-6215-13-229 (PMC3543249; doi:10.1186/1745-6215-13-229)
Supplement: Additional file 2 — Set of Process and outcome indicators for in-hospital management of COPD exacerbation. This Additional file displays a set of validated process and outcome indicators for audit of care for in-hospital management of COPD exacerbation. [file 1745-6215-13-229-S2.pdf]

Additional file 2: Set of process and outcome indicators for in-hospital management of COPD exacerbation

Process indicators	Typology	Criterion met	Unit
1. Performance of arterial blood gas (ABG) measurement during first 24 hours of admission	Process	Performed in all patients	Percentage
2. Performance of chest X-ray during first 24 hours of admission[†]	Process	Performed in all patients	Percentage
3. Performance of electrocardiogram during first 24 hours of admission[†]	Process	Performed in all patients	Percentage
4. Performance of sputum culture and antibiogram during hospitalisation (additional information)	Process	Differences in rates	Percentage
5. Measurement of FEV1 during hospitalisation	Process	Differences in rates	Percentage
6. Prescription of short acting bronchodilators	Process	Performed in all patients	Percentage
7. Prescription of long-acting bronchodilators	Process	Performed in all patients	Percentage
8. Adequate prescription of systemic glucocorticoids[†]	Process	Performed in all patients	Percentage
9. Prescription of antibiotics (additional information)	Process	Performed in all patients	Percentage
10. Administration of controlled oxygen therapy in patients hypoxaemic during admission[†]	Process	Performed in all patients	Percentage
11. Assessment of smoking status at admission	Process	Performed in all patients	Percentage
12. Smoking cessation intervention in active smokers at admission[†]	Process	Performed in all patients	Percentage
13. Adequate education regarding inhaler therapy	Process	Performed in all patients	Percentage
14. Adequate education regarding home oxygen therapy[†]	Process	Performed in all patients	Percentage
15. Performance of rehabilitation tests during the past year	Process	Performed in all patients	Percentage
16. Referral to pulmonary rehabilitation during the past year[†]	Process	Differences in rates	Percentage
17. Assessment of nutritional status	Process	Performed in all patients	Percentage
18. Adequate management of underweight[†]	Process	Performed in all patients	Percentage
19. Adequate management of overweight[†]	Process	Performed in all patients	Percentage
20. Screening and updating of influenza vaccination	Process	Performed in all patients	Percentage
21. Screening and updating of pneumococcal vaccination	Process	Performed in all patients	Percentage
22. Measurement of ABGs 1 or 2 days before discharge inpatients hypoxemic at admission	Process	Performed in all patients	Percentage
23. Prescription of home oxygen therapy in patients hypoxaemic at discharge[†]	Process	Performed in all patients	Percentage
24. Adequate discharge management[†]	Process	Performed in all patients	Percentage
**Outcome indicators**	**Typology**	**Criterion met**	**Unit**
1. Readmission: 30-day, 6-month	Outcome	Differences in rates	Percentage
2. Mortality: In-hospital, 30-day, 6-month	Outcome	Differences in rates	Percentage
3. Length of stay (LOS)	Outcome	Differences in values	Days
4. Level of understanding of inhaler therapy	Outcome	Differences in values	Score
5. Compliance with home oxygen therapy 30 days after discharge[a]	Outcome	Differences in values	Score
6. Performance of physical exercise during 30 days after discharge	Outcome	Differences in values	Score
7. Smoking status: 30 days after discharge, 6 months after discharge	Outcome	Differences in rates	Percentage
8. Symptoms of anxiety and depression: at discharge, 30 days after discharge *Hospital Anxiety and Depression Scale (HADS) [1]*	Outcome	Differences in rates	Percentage
9. Health-related quality of life (HRQL) 30 days after discharge *Saint George Respiratory Questionnaire (SGRQ)[2]*	Outcome	Differences in values	Score
10. Able to cope at home/place of residence at 30 days after discharge	Outcome	Differences in values	Score
11. Functional status: at discharge, 30 days and 6 months after discharge *Katz Scale [3]*	Outcome	Differences in rates	Percentage
12. Self-reported health condition: at discharge, 30 days and 6 months after discharge	Outcome	Differences in values	Score
13. Severity of breathlessness at 30 days after discharge Modified Medical Research Council Dyspnoea scale [4]	Outcome	Differences in values	Score

14. Number of visits by general practitioner at 30 days after discharge	Outcome	Differences in values	Score
15. Health status http://www.euroqol.org/	Outcome	Used for economic analyses	Score
Baseline variables<br>• *Socioeconomic variables:* income, medical insurance, level of education, profession<br>• *Demographic variables:* age, gender, nationality<br>• *COPD-related variables:* COPD severity, comorbidities, cognitive status, smoking status at admission, COPD-related hospitalisation in the previous year, medication prescribed before hospitalisation, use of home oxygen therapy before admission, body mass index, lung function parameters before discharge, arterial blood gas values at admission, non-invasive positive pressure ventilation (NIPPV)			

[†]Multi-component indicators.

1. Zigmond AS, Snaith RP: **The hospital anxiety and depression scale.** *Acta Psychiatr Scand* 1983, 67:361-70.
2. Jones PW, Quirk FH, Baveystock CM. The St George's Respiratory Questionnaire. *Respir Med* 1991,**85**:25-31.
3. Katz S, Ford AB, Moskowitz RW, Jackson BA, Jaffe MW. **Studies of illness in the aged. The indes of ADL: a standardized measure of biological and psychosocial function.** *JAMA* 1963,**185**:914-9.
4. Bestall JC, Paul EA, Garrod R, Garnham R, Jones PW, Wedzicha JA. **Usefulness of the Medical Research Council (MRC) dyspnoea scale as a measure of disability in patients with chronic obstructive pulmonary disease.** *Thorax* 1999,**54**:581-6.
