# Supplementary material for: Eight-step method to build the clinical content of an evidence-based care pathway: the case for COPD exacerbation
Source: Trials. 2012 Nov 29;13:229. doi: 10.1186/1745-6215-13-229 (PMC3543249; doi:10.1186/1745-6215-13-229)
Supplement: Additional file 3 — Example of description of an indicator. This Additional file displays the detailed description of an indicator according to the guidance of the Agency for Health Care Research and Quality (http://www.qualitymeasures.ahrq.gov) and the Joint Commission (http://www.jointcommission.org). [file 1745-6215-13-229-S3.pdf]

Additional file 3. Example of description of an indicator

**Indicator number**	**2.1.**
**Indicator name**	**Arterial blood gas (ABG) measurement at admission**
**Description**	At admission, measurement of PaO_2, $PaCO_2$, $H_2CO_3^-$, SaO_2, and pH by arterial puncture (radialis, brachialis, or femoralis), while breathing room air in patients admitted with the principal diagnosis ICD-9-CM code 491.21.<br>If measurement of ABGs while breathing room air is not feasible (severe cases), oxygen flow (l/min) should be noted. Twenty to 30 minutes should pass before rechecking the ABG tensions when the FiO_2 has been changed.
**Rationale/ Relation to quality**	Blood gas monitoring is mandatory for patients that require hospitalisation during acute exacerbations of COPD, as measurement of ABGs is important to assess the severity of an exacerbation. A PaO_2 <8.0 kPa (60 mm Hg) and/or SaO_2 <90% with or without $PaCO_2$ >6.7 kPa (50 mmHg) when breathing room air indicate respiratory failure. In addition, moderate-to-severe acidosis (pH <7.36) plus hypercapnia ($PaCO_2$ >6-8 kPa; 45-60 mmHg) in a patient with respiratory failure is an indication for mechanical ventilation. Since arterial blood pH is usually relatively normal in stable COPD, its value during an hypercapnic exacerbation is a useful index of the acute rise in $PaCO_2$, and in turn, is also related to the prognosis of the exacerbation. In conclusion, failing to obtain patients' ABGs may lead to suboptimal management, as ABG values are an important determinant for initiating supplemental oxygen therapy, for prescribing assisted ventilation, and for prescribing home oxygen therapy. Furthermore, patients presenting with exacerbations often suffer from hypercapnia, which can only be detected through ABG measurement. Studies point out that performance of ABGs range from 44% to 84%, with a variance across hospitals of more than 20%.
**Type of indicator**	Process
**Numerator**	Number of patients in which ABG measurement at admission was performed among cases meeting the inclusion and exclusion criteria for the denominator among cases meeting the inclusion and exclusion criteria for the denominator.
**Denominator**	Total number of patients discharged with a principal diagnosis ICD-9-CM code 491.21 as defined in *Preface Table 1.*<br>**Inclusion criteria:** all inclusion criteria defined in *Preface Table 2.*<br>**Exclusion criteria:** all exclusion criteria defined in *Preface Table 2.*
**Data collection method**	**Retrospective: Patient record**<br>Who: External researcher<br>Time point: After discharge from the ward<br>Data:<br>• ABG measurement within first 24 hours of admission? (ABGAD)<br>*Data for check:*<br>• *Date of admission (PRADDate)*<br>• *Date of first ABG measurements at admission (ABGADDa)*<br>• *ABG values (PaO_2, $PaCO_2$, pH, SaO_2, H_2CO_3) measured at times 1, 2, and 3*
**Data elements for indicator**	• ABG measurement at day of admission? (ABGAD)
**Data reported as**	Aggregate rate (%) generated form count data (n) reported as proportion (n/n)
**Expected outcome**	44-84%
**Criteria to meet**	Performed in all patients (100%)
**References:**	(Barbera et al., 1997; Bratzler et al., 2004; Calverley, 2000; Celli & Macnee, 2004; Chang et al., 2007; Cydulka et al., 2003; Gibson & Macnee, 2008; Global Initiative for Chronic Obstructive Lung Disease, 2008a; Harvey et al., 2005; Kelly & Elborn, 2002; Lindenauer et al., 2006b; Roberts et al., 2001; Rodriguez-Roisin, 2006a; Siafkas & Wedzicha, 2006)
